# Anti-N-Methyl-D-Aspartate Receptor Encephalitis: A New Challenging Entity for Consultation-Liaison Psychiatrist

**Published:** 2016-05-10

**Authors:** GE Maccaferri, AO Rossetti, J Dalmau, A Berney

**Affiliations:** 1 Psychiatric Liaison Service, Lausanne University Hospital (CHUV), Switzerland; 2 Department of Clinical Neurosciences, Lausanne University Hospital (CHUV), Switzerland; 3 August Pi i Sunyer Biomedical Research Institute (IDIBAPS), Service of Neurology, Hospital Clinic, University of Barcelona, Spain; 4 Catalan Institution for Research and Advanced Studies (ICREA), Barcelona, Spain; 5 Department of Neurology, University of Pennsylvania, PA, USA

**Keywords:** Autoimmune, Anti-N-methyl-D-aspartate receptor, Encephalitis, Organic psychosis, Rehabilitation

## Abstract

**Background:**

Anti-N-methyl-D-aspartate receptor (anti-NMDAR) encephalitis is a relatively newly identified autoimmune neuropsychiatric disorder that predominantly affects children and young adults. Although psychiatric symptoms are highly prevalent and frequently severe, it has mainly been reported in neurological, but not psychiatric, literature. Understanding this form of encephalitis, its quick diagnosis and which treatment to provide are of utmost importance for consultation-liaison (C-L) psychiatrists. The aim of this paper was to describe a case of anti-NMDAR encephalitis with severe psychiatric manifestations, who showed impressive recovery but required intensive involvement of the C-L psychiatry team. We emphasise the behavioural aspects, psychiatric symptoms and challenges faced by the CL consultant across the different phases of the treatment.

**Methods:**

We report the different treatment phases for a young woman with anti-NMDAR encephalitis who developed severe neuropsychiatric symptoms, with a focus on the role and challenges faced by the C-L psychiatrist. The literature is reviewed for each of these challenges.

**Results:**

This case illustrated that even extremely severely affected patients may show impressive recovery, but require long lasting psychiatric care. C-L psychiatrists are faced with numerous challenges where only little literature is available.

**Conclusion:**

C-L psychiatrists play a pivotal role throughout the multidisciplinary care of patients with anti-NMDAR encephalitis and should be informed about this entity.

## Introduction

Anti-N-methyl-D-aspartate receptor (anti-NMDAR) encephalitis is a subtype of a recently described autoimmune disorder of the brain. It was first described in 2005 and subsequently has been characterized mainly in the neurological literature [1,2]. There are surprisingly few reports in psychiatric journals, given that patients with anti-NMDAR encephalitis often present with psychiatric symptoms including psychomotor agitation, impulsivity and disinhibition, mood swings, delusional thoughts, paranoia, and hallucinations [3]. Up to 25% of patients may have a poor outcome, with persistent, severe neuropsychiatric deficits or even die [2,4]. Understanding this entity, performing a quick diagnosis and deciding treatment are important for consultation-liaison (C-L) psychiatrists.

In this paper we illustrate the challenges faced by the C-L consultant across different phases of the treatment of a young woman with severe encephalitis, who displayed cognitive behavioural and psychiatric symptoms but nevertheless impressively recovered after a very long-lasting disease course.

## Case Illustration

### Prodromal phase

**Figure 1:**
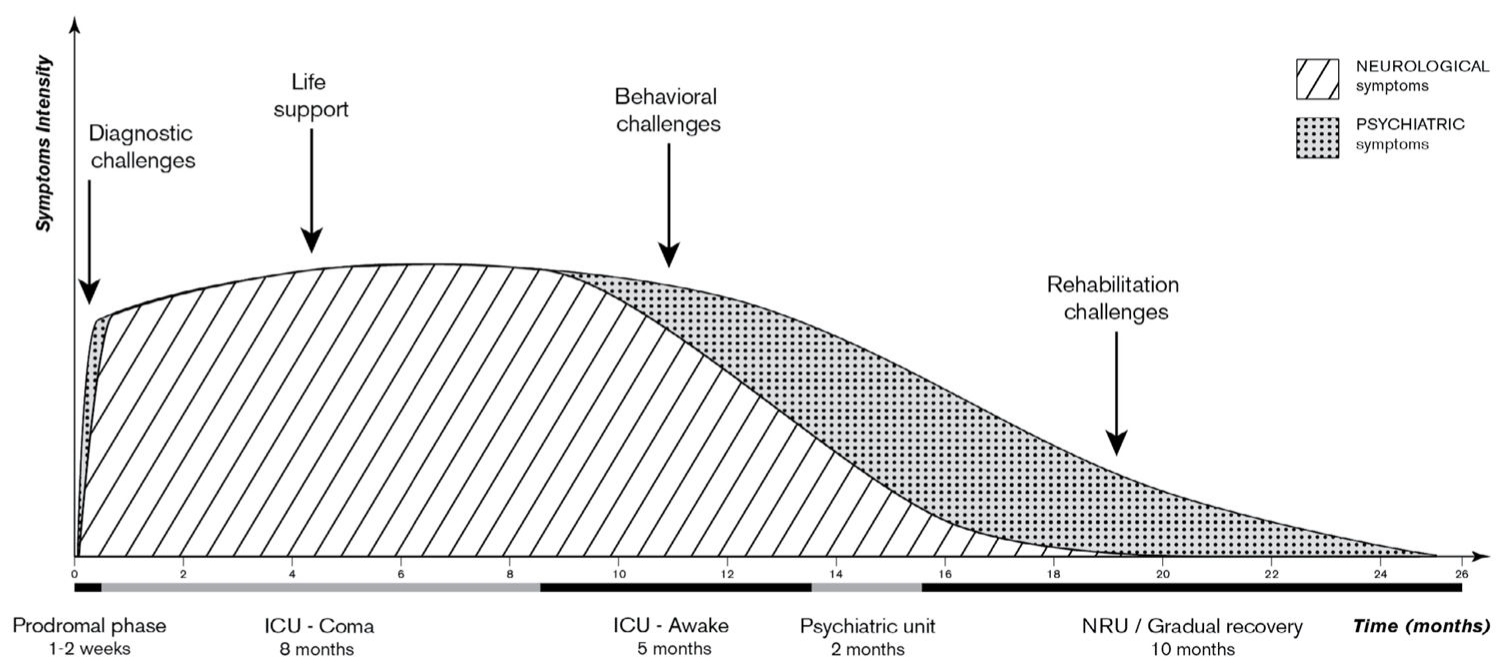
Intensity and duration of neurological and psychiatric symptoms through the different phases of the anti-NMDAR encephalitis. **Note:** ICU, intensive care unit; NRU, neuro rehabilitation unit; Anti-NMDAR, anti-N-methyl-D-aspartate receptor **Course of neurological symptoms:** abnormal movements, paraesthesia, swallowing disorders, speech deterioration, delirium, autonomic dysregulation, coma, and gradual recovery. **Course of psychiatric symptoms:** mental fatigue, concentration difficulties, psychotic symptoms, delirium, coma, acute confusional state, severe behavioural disorders (psychomotor agitation, psychotic symptoms, suicidal ideation, irritability and emotional manifestations towards caregivers), residual psychotic symptoms (affective flattening, apathy and social withdrawal sadness, anger, fluctuating suicidal ideation), and gradual recovery.

A 22-year-old female university student with no medical and psychiatric history spontaneously presented to the emergency unit of our university hospital, complaining of speech disorders, physical and mental fatigue. Four days earlier she had developed a stuttering episode with concentration difficulties. A side effect of a recently prescribed antihistaminic drug (cetirizine) was suspected, and the medication was stopped. She was discharged home. Two days later, she was readmitted to the hospital because of an increase in subtle chorei form movements (perioral, hands), as well as memory impairment. She also presented with decreased mobility, lack of right hand grip, paraesthesia of the left hemibody, dysarthria, and tinnitus. Figure 1 illustrates the intensity of neurological and psychiatric symptoms throughout the different phases of the disease.

### First intensive care unit (ICU) phase: Coma

In the following days, her orofacial dyskinesia worsened and she developed a swallowing disorder, speech deterioration, vomiting, wide pupils and facial flush, right upper limb weakness associated with paranoid delusions, as well as auditory and olfactory hallucinations. Over the following 2 weeks she developed delirium and increased impairment of consciousness, requiring endotracheal intubation and ventilation in the ICU. A large differential diagnosis was considered, including most common causes of encephalitis: viral, autoimmune, paraneoplastic and toxic-metabolic. Electroencephalogram (EEG) performed shortly after inpatient admission (2 weeks after the initial symptoms) was suggestive of anti-NMDAR encephalitis, showing poorly reactive rhythmic delta activity over the frontal regions [5]. This was confirmed by a positive anti-NMDAR antibody test in cerebrospinal fluid and serum (Serum: 1/100, Cerebrospinal fluid: 1/2). A nodule in the right ovary observed by ultrasound and removed by laparoscopy did not show a tumoural lesion by histology (no teratoma).

During the following months, the patient, while unconscious and mechanically ventilated, received specific immunological treatments (high-dose steroids, 15 cycles of plasma exchange, 8 cycles of cyclophosphamide, 4 cycles of rituximab, and 4 cycles of intravenous immunoglobulin), with high-dose midazolam (and at times propofol) sedation. Although we did not formally identify a clinical or electrographic seizure, she received non-sedating intravenous antiepileptic treatment (valproic acid and lacosamide), because the rhythmic delta activity by EEG represented an ictal correlate [6]. Her clinical course was characterized by hemodynamic, breathing, cardiac arrhythmias, infectious, metabolic, and nutritional dysfunctions. Throughout this long “unresponsive phase” there was no contact with the patient. Sometimes she opened her eyes but remained mute and unresponsive to visual stimuli. Orofacial and arm dyskinesia were regularly present upon weaning of the sedation, and her EEG showed a rhythm delta over the frontal regions alternating with a disorganized, relatively fast activity.

### Second ICU phase: Awake

After several months of being in an unresponsive state in the ICU under continuous immunotherapy, the patient started to improve. Limited contact became possible: she opened her eyes and responded to simple orders. She was finally extubated eight months after her admission. After awakening, the patient presented with an acute confusional state but her neurological status improved slowly. Over the following weeks, she was slow and hypomimic with ataxic movements. However, delirium persisted with episodes where she was mute, a kinetic, and unresponsive to verbal commands. For four months the patient presented with severe psychopathological manifestations.

1. **Episodes of psychomotor agitation** with overt aggressive behaviour were very challenging, including throwing objects around the room, slapping her hands on the bed, trying to pull out the feeding tube, sometimes requiring sedation (intravenous midazolam and propofol) and even physical restraint for short periods.
2. **Psychotic features** were clearly recognizable upon speaking indicating paranoid delusions as well as auditory and olfactory hallucinations. She was also tremendously anxious or perplexed, and refused contact (e.g. actively kept her eyes closed) with the ICU staff or even with her family.
3. **Affective elements** were also present, including depressive withdrawal, suicidal ideation culminating in attempted self-harm by knife or strangulation with a cable. She also presented with severe insomnia and nightmares.
4. **Interactions with her caregivers** were marked by irritability and emotional manifestations, recurrent refusal of care and feeding, and even requests to be killed or that we let her die. This highly challenging situation for the ICU team required that an additional nurse be hired allowing “one to one” continuous supervision of the patient to prevent accidents.

Besides midazolam and propofol she received quetiapine XR (orally through a nasogastric tube). Despite benzodiazepine and antipsychotic treatments, the patient remained impulsive and aggressive with psychotic symptoms for several weeks. An improvement occurred after 1 month when quetiapine XR was increased to 600 mg/day. In parallel, physiotherapy was started in the ICU to improve swallowing and reduce feeding difficulties. A specific team (not the C-L team) delivered these therapies. The neuropsychological rehabilitation therapies were used to target attention disorders, immediate and episodic anterograde memory, and language disorders such as dysarthria, lack of words and ideomotor apraxia.

Four months after coma resolution (12 months after admission), we observed a significant improvement that was chronologically linked to a decrease in plasma anti-NMDAR antibodies. A further increase of quetiapine allowed a low tapering of benzodiazepines, at which time, there was a complete disappearance of psychomotor agitation. However, hallucinations as well as feelings of sadness, anger, and fluctuating suicidal ideation were still present.

### Rehabilitation phase

#### Residual psychotic and affective symptoms

Five months after coma resolution, the patient was transferred to a psychiatric unit for management and treatment of the residual psychotic symptoms. Her autonomy increased and she better engaged in social interactions with therapists and other patients. She was collaborative in psychiatric consultations, physiotherapy and occupational therapy activities. She began to take better care of her body, applying make-up or asking for an appointment with the hairdresser. Contacts with family and friends were more frequent and a source of pleasure. She started to make plans for her future (wish to be discharged home and return to her university studies). After 2 months at the psychiatric unit, the psychotic and affective symptoms gradually resolved.

#### Reversible cognitive impairment

At that point, cognitive deficits were still present, with attention disorders, deficits in immediate and episodic anterograde memory, language disorders (dysarthria, lack of words, paraphasia and agrammatism), and ideomotor apraxia. The patient was transferred to a neurorehabilitation unit (NRU) for about 6 weeks. She was trained by neuropsychological tasks and showed improved interference management, information retention in episodic and verbal memory, and mental calculations. Furthermore, she benefited from intensive ergotherapeutic and physiotherapeutic approaches. In particular, ergotherapy by NRU staff focused on cognitive, speech-language deficits, communication skills and social-motivational occupations. The clinical evolution was remarkably good, with an improvement in cognitive and executive functions and social organizational activities.

#### Gradual recovery

Sixteen months after her first contact with our hospital, the patient was discharged home. She was followed up by liaison psychiatry consultation once a week. She took quetiapine (300 mg/day) treatment for 6 more months, which was then successfully discontinued. Nine months after discharge, the patient and her parents reported social, personal and family stability. The patient resumed university attendance and successfully passed her exams. She has little recollection of the 16 months of admission and treatment, particularly the ICU phase. At the last follow-up, 2 years after the event, she only complained about the tracheostomy scar.

## Discussion

This study reports the case of a young woman presenting with anti-NMDAR encephalitis, an autoimmune and paraneoplastic disorder that affects young adults and may be associated with tumours (ovarian teratoma) in up to 50% of cases [2]. Anti-NMDAR encephalitis has been recently described following the ability to measure this antibody. Most patients develop a complex syndrome, initially presenting with psychosis and behavioural disturbances and rapidly progressing to develop speech problems, memory deficits, consciousness impairment, movement disorders, seizures, dysautonomia, and respiratory deregulation. Although some authors describe very distinct clinical phases [7], an exact sequential development of stages is not always observed. For example, the presenting symptoms (e.g. more frequent psychiatric episodes in younger adults and teenagers, more frequent seizures or dyskinesias in young children), development of a full blown syndrome, or clinical course and relapses can vary among patients [8–11]. Furthermore, a recent study highlighted the possibility of isolated psychiatric presentation (without neurological symptoms) [12], and Soe et al. recently described the first case of β-Thalassemia trait association with anti-NMDAR encephalitis in a 10-year-old girl [13]. Our case illustrates that even extremely severely affected patients may show impressive recovery but require long-term psychiatric care. C-L psychiatrists are faced with numerous challenges (Figure 1) because of a lack of available literature.

The care of our patient required the involvement of numerous disciplines as well as several medical structures (ICU, acute neurology, NRU, psychiatric facilities). The special position of the C-L consultant not only required collaboration with all these disciplines but also provided continuity throughout the entire period of care. This aspect appeared to be of utmost importance to the patient at several time points. Our role with ICU or NRU staff included education, support, managing counter transference to the patient and C-L team, advice on pharmacotherapy and behavioural approaches.

Here we focus on the main challenges and strategies employed by the C-L psychiatrist (Figure 1).

## Diagnostic Challenges

In many cases of anti-NMDAR encephalitis, especially early in the course of the disease, symptoms and behaviours mimicking psychotic or mood/anxiety disorders, such as hallucinations, delusions, anxiety, depressive symptoms and fatigue are observed. In our patient, the rapid progression of mental fatigue and concentration difficulties to psychotic symptoms and auditory and olfactory hallucinations together with neurological impairment (delirium, abnormal movements, speech deterioration) were highly suggestive of an organic cause, rather than a primary psychiatric disorder. Important differential diagnoses apart from encephalitis, included serotonin and neuroleptic malignant syndromes given that neurological symptoms (hyperreflexia, myoclonus, restless syndrome, rigidity, behavioural disorders) and delirium can be present in both [4,14]. Tricyclics, selective serotonin reuptake inhibitors, antidepressants, and neuroleptics may induce autonomic dysregulation as well as hyperreflexia, myoclonus, restless leg syndrome, rigidity, behavioural disorders and delirium. Without treatment, the clinical picture can evolve into malignant hyperthermia, muscular rigidity, dyskinesia progressing to unstable blood pressure and autonomic dysregulation [4,14]. In our case, a detailed history provided by her family and treating physician allowed the exclusion of antipsychotic and/or antidepressant use before symptom onset. Based on this clinical presentation and the EEG, a suspicion of anti-NMDAR encephalitis was finally confirmed by blood test.

## Behavioural Challenges

Our C-L psychiatric team had to face a patient with severe psychomotor agitation, aggressive behaviour, psychotic manifestations, suicidal ideation and irritability towards caregivers and her family for many months. Pharmacological and non-pharmacological strategies were applied.

### Pharmacological Strategies

In our case, suppression of the immune response improved neurological signs slowly but initially had no impact on psychiatric symptoms. The patient was easier to manage when sedated in the ICU, compared with the second period in the ICU when she awoke and had marked psychiatric symptoms (Figure 1). Literature for the pharmacological management of psychiatric and behaviour symptoms in the context of anti-NMDAR encephalitis is sparse but suggests the use of atypical (olanzapine, quetiapine, risperidone, ziprasidone) antipsychotics and in extreme conditions, phenobarbital, benzodiazepines, or fentanyl, to induce a medical coma [12,15]. All antipsychotics must be employed with caution because they can exacerbate motor symptoms, worsen a malignant catatonia (a possible later complication induced by encephalitis progression), or trigger neuroleptic malignant syndrome [4,16]. Benzodiazepines (lorazepam) can be effective in cases of catatonia, but few published reports describe the use of electroconvulsive therapy if catatonia is benzodiazepines-resistant [15,17,18]. Anecdotal case reports show that lithium and valproic acid use can treat mood dysregulation symptoms in anti-NMDAR encephalitis [19,20]. In our case, the use of quetiapine up to 600 mg/day after 3–4 weeks coupled with high doses of benzodiazepines (midazolam 4 mg i.v. per hour) and Propofol to treat agitation was an efficacious strategy. We faced a risk/benefit dilemma between the need for sedation and the risk of triggering or perpetuating delirium. Midazolam and propofol are potent sedative agents but are delirium risk factors. These treatments were initiated by ICU physicians. The role of the C-L psychiatrist was to frequently remind the ICU of this risk and try to optimize the risk/benefit for the patient. Each drug administration and increase in benzodiazepine or neuroleptic dosage was carefully monitored. We prescribed the lowest possible dose with optimal distribution over the day, and avoided polypharmacy interactions. We measured cardiac QT interval to evaluate and limit the risk of arrhythmia, “torsade de pointes” and the risk of sudden death [21,22]. Furthermore, to optimize the treatment, we conducted frequent therapeutic drug monitoring (TDM) of psychotropic drugs based on the Arbeitsgemeinschaft für Neuropsychopharmakologie und Pharmakopsychiatrie (Association for Neuropsychopharmacology and Pharmacopsychiatry) guidelines for TDM in psychiatry [23,24]. We followed the recommendations for drug concentrations and laboratory alert levels. We also obtained advice from the Psychopharmacology Unit of our University Hospital.

### Non-pharmacological strategies

We found no studies examining the role of non-pharmacological interventions to treat behavioural disorders in the context of anti-NMDAR encephalitis. In our case, the main objective of the medical team was to ensure the safety of the patient and reduce psychomotor agitation. We faced some extreme violent behaviour as well as treatment-resistant visual, auditory and olfactory hallucinations during several weeks.

General hospitals in Switzerland use written protocols for authorizing and providing physical or chemical restraint. These protocols are prescribed by the physician in charge with the advice and supervision of the C-L psychiatric team. The duration must be strictly limited. In our case, restraint was applied for the shortest time possible and only at times when the patient had low discernment capacity. When restraint was applied, it was discussed at least twice a day with the team and monitored according to a formal written protocol regulating physical restraint. Throughout the difficult phases we took great care to try to reorient the patient, to explain the context of care, and answer her questions. During the prolonged stay in the ICU, our role with medical staff included support and managing countertransference. In particular, the negative attitudes of the team expressed through aggressive comments and disinvestment in the care of the patient were discussed in multidisciplinary team meetings.

## Rehabilitation Challenges

Five months after coma resolution, the patient was admitted to the psychiatric unit for 2 months, as psychiatric symptoms were still too severe to follow up directly with a rehabilitation program. Transfer to psychiatric hospital was also discussed at several multidisciplinary meetings. Moreover, the psychiatric hospital team representative discussed the transfer to ensure a smooth transition between the somatic and psychiatric ward.

Throughout the psychiatric unit period, residual psychotic symptoms (affective flattening, apathy and social withdrawal) were the new focus of treatment. Frequent psychiatric consultations, occupational therapy activities, and contact with her family provided a good psychiatric outcome.

Our patient was then transferred to the NRU for about 6 weeks. There is limited literature for the long-term rehabilitation of patients with anti-NMDAR encephalitis. A recent clinical case conference demonstrated the necessity of individualizes treatments and targeting different strategies depending on the type of clinical presentation [20]. The authors suggested combining physical and occupational therapies to target different deficits after a long hospital stay. According to the type of deficit, different clinical techniques are employed including speech-language work, re-feeding strategies after prolonged ventilation, physiotherapy for the recovery of coordination and simple walking. For memory impairment, a recent report reported long-term cognitive outcomes in nine patients with anti-NMDAR encephalitis [25]. The authors observed that cognitive deficits (mainly impairments in executive functions and memory) may be present for a long period after resolution of the acute stage of anti-NMDAR encephalitis. Furthermore, the severity of cognitive impairment showed inter-individual variation and depended on disease course and treatment onset. This suggests the importance of early and aggressive treatment by immunotherapy to limit or prevent a later cognitive impairment. In our patient, treatment targeted attention disorders, immediate and episodic anterograde memory, language disorders as dysarthria, lack of words and ideomotor apraxia. Ergotherapy focused on cognitive, speech-language deficits, communication skills and social-motivational occupations. Physiotherapy was useful to provide better coordination. After discharge from the NRU, the patient was followed up for a couple of months by our C-L psychiatric outpatient unit. The main goal was to allow verbalization of emotional experiences such as the fear of death and of a relapse. Given the limited recollection of the 16-month hospital admission, the patient was in need of retracing the story of the different phases of the disease: this step allowed a reappropriation of this dramatic episode of her life. Moreover, the patient was in need of support to regain her desire for emancipation as a young adult woman who is responsible for her future.

## Conclusion

Anti-NMDAR encephalitis is a newly described pathology at the interface between neurology and psychiatry. It is important to improve the knowledge of C-L psychiatrists with regard to this organic condition that frequently presents with severe psychiatric symptoms. Multidisciplinary collaboration is important throughout the different phases of this disease.

## References

[1] Vitaliani R, Mason W, Ances B, Zwerdling T, Jiang Z (2005). Paraneoplastic encephalitis, psychiatric symptoms, and hypoventilation in ovarian teratoma. Ann Neurol.

[2] Dalmau J, Gleichman AJ, Hughes EG, Rossi JE, Peng X (2008). Anti-NMDA-receptor encephalitis: case series and analysis of the effects of antibodies. Lancet Neurol.

[3] Maneta E, Garcia G (2014). Psychiatric manifestations of anti-NMDA receptor encephalitis: neurobiological underpinnings and differential diagnostic implications. Psychosomatics.

[4] Dalmau J, Lancaster E, Martinez-Hernandez E, Rosenfeld MR, Balice-Gordon R (2011). Clinical experience and laboratory investigations in patients with anti-NMDAR encephalitis. Lancet Neurol.

[5] Nosadini M, Boniver C, Zuliani L, de Palma L, Cainelli E (2015). Longitudinal electroencephalographic (EEG) findings in pediatric anti-N-methyl-D-aspartate (anti-NMDA) receptor encephalitis: the Padua experience. J Child Neurol.

[6] Kirkpatrick MP, Clarke CD, Sonmezturk HH, Abou-Khalil B (2011). Rhythmic delta activity represents a form of nonconvulsive status epilepticus in anti-NMDA receptor antibody encephalitis. Epilepsy Behav.

[7] Iizuka T, Sakai F, Ide T, Monzen T, Yoshii S (2008). Anti-NMDA receptor encephalitis in Japan: long-term outcome without tumor removal. Neurology.

[8] Armangue T, Titulaer MJ, Málaga I, Bataller L, Gabilondo I (2013). Pediatric anti-N-methyl-D-aspartate receptor encephalitis-clinical analysis and novel findings in a series of 20 patients. J Pediatr.

[9] Titulaer MJ, McCracken L, Gabilondo I, Armangué T, Glaser C (2013). Treatment and prognostic factors for long-term outcome in patients with anti-NMDA receptor encephalitis: an observational cohort study. Lancet Neurol.

[10] Titulaer MJ, McCracken L, Gabilondo I, Iizuka T, Kawachi I (2013). Late-onset anti-NMDA receptor encephalitis. Neurology.

[11] Yuan N, Glezer A (2013). A young woman presenting with psychotic and mood symptoms from anti-N-methyl-D-aspartate receptor (NMDA-R) encephalitis: an emerging diagnosis. Int J Psychiatry Med.

[12] Kayser MS, Dalmau J (2011). Anti-NMDA Receptor Encephalitis in Psychiatry. Curr Psychiatry Rev.

[13] Soe K, Kowalik J, La Rosa A, Caplan JP (2016). β-Thalassemia Trait Association With Anti-N-methyl-d-aspartate Receptor Encephalitis: Literature Review and Case Report. Psychosomatics.

[14] Barry H, Hardiman O, Healy DG, Keogan M, Moroney J (2011). Anti-NMDA receptor encephalitis: an important differential diagnosis in psychosis. Br J Psychiatry.

[15] Florance NR, Davis RL, Lam C, Szperka C, Zhou L (2009). Anti-N-methyl-D-aspartate receptor (NMDAR) encephalitis in children and adolescents. Ann Neurol.

[16] Fink M, Taylor MA (2009). The catatonia syndrome: forgotten but not gone. Arch Gen Psychiatry.

[17] Braakman HM, Moers-Hornikx VM, Arts BM, Hupperts RM, Nicolai J (2010). Pearls & Oy-sters: electroconvulsive therapy in anti-NMDA receptor encephalitis. Neurology.

[18] Lee A, Glick DB, Dinwiddie SH (2006). Electroconvulsive therapy in a pediatric patient with malignant catatonia and paraneoplastic limbic encephalitis. J ECT.

[19] Kayser MS, Titulaer MJ, Gresa-Arribas N, Dalmau J (2013). Frequency and characteristics of isolated psychiatric episodes in anti-N-methyl-d-aspartate receptor encephalitis. JAMA Neurol.

[20] Chapman MR, Vause HE (2011). Anti-NMDA receptor encephalitis: diagnosis, psychiatric presentation, and treatment. Am J Psychiatry.

[21] Haddad PM, Anderson IM (2002). Antipsychotic-related QTc prolongation, torsade de pointes and sudden death. Drugs.

[22] Malik M, Camm AJ (2001). Evaluation of drug-induced QT interval prolongation: implications for drug approval and labelling. Drug Saf.

[23] Hiemke C, Baumann P, Bergemann N, Conca A, Dietmaier O (2011). AGNP consensus guidelines for therapeutic drug monitoring in psychiatry: Update 2011. Pharmacopsychiatry.

[24] Sparshatt A, Taylor D, Patel MX, Kapur S (2011). Relationship between daily dose, plasma concentrations, dopamine receptor occupancy, and clinical response to quetiapine: a review. J Clin Psychiatry.

[25] Finke C, Kopp UA, Prüss H, Dalmau J, Wandinger KP (2012). Cognitive deficits following anti-NMDA receptor encephalitis. J Neurol Neurosurg Psychiatry.

